# Study of the NLRP3 inflammasome component genes and downstream cytokines in patients with type 2 diabetes mellitus with carotid atherosclerosis

**DOI:** 10.1186/s12944-017-0595-2

**Published:** 2017-11-18

**Authors:** Junli Lee, Jing Wan, Linyun Lee, Changhua Peng, Hailong Xie, Chengbin Lee

**Affiliations:** 1 Department of Clinical Laboratory, The second clinical medical college of yangtze university, Ren Min Road 1#, Jingzhou, Hubei 434020 China; 2 Department of Endocrinology, The second clinical medical college of yangtze university, Jingzhou, China; 3 Department of Clinical Medicine, Graduate School of Yangtze University, Jingzhou, China

**Keywords:** NLRP3, Cytokines,type 2 diabetes mellitus,carotid atherosclerosis

## Abstract

**Background:**

A role for the NLRP3 inflammasome has been reported in various diseases, such as diabetes mellitus, atherosclerosis (AS), nephropathy, rheumatism, and others, although limited information is available concerning the role of the NLRP3 inflammasome, interleukin-1β (IL-1β) and interleukin-18 (IL-18) in patients with type 2 diabetes mellitus (T2DM) and carotid atherosclerosis (CAS). Therefore, this cross-sectional study investigated these inflammatory components in patients with T2DM complicated with carotid atherosclerosis (T2DM + CAS).

**Methods:**

A total of 107 inpatients or outpatients were included,including 81 T2DM + CAS patients and 26 T2DM patients. Patients with T2DM or T2DM + CAS were recruited to compare the expression levels of NLRP3 pathway genes (NLRP3, ASC and caspase-1 mRNA) and the serum IL-1β and IL-18 concentrations. In the T2DM + CAS group, patients with thickened intima media thickness (IMT) and those with plaques were compared, and the correlation of the 5 variables with Crouse scores were analyzed.

**Results:**

The expression of NLRP3 pathway genes except caspase-1 was significantly higher in patients with T2DM and CAS compared to T2DM patients. Serum IL-1β and IL-18 concentrations shows no difference between the T2DM + CAS and T2DM group. In the T2DM + CAS group, the expression levels of the three inflammasome genes and IL-18 were increased in patients with thickened IMT compared to those with the plaque. All of the above factors negatively correlated with Crouse scores.

**Conclusion:**

NLRP3 inflammasome pathway activity is significantly increased in patients with AS and T2DM at the early stage of plaque formation.

## Background

Diabetes mellitus is the second-leading cause of death among contemporary diseases. Vascular lesions, a complication of diabetes mellitus leading to high mortality, occur as a result of atherosclerosis (AS). AS and type 2 diabetes mellitus (T2DM) are metabolic diseases and share similar pathophysiological mechanisms, including NLRP3 (nucleotide-binding domain, leucine-rich-containing family, pyrin domain-containing-3) inflammasome activation, which has been highlighted in recent studies [1, 2]. The NLRP3 inflammasome is the most well-studied inflammasome identified thus far; this cytosolic protein complex includes NLRP3, ASC (apoptosis-associated speck-like protein containing a CARD) and caspase-1 [3]. NLRP3 serves as the sensor of inflammasome activation, which can be triggered via cholesterol crystals, uric acid, microorganisms, and a variety of other ligands [4, 5]. Following recognition of the corresponding ligand by NLRP3, the adaptor protein ASC is clustered to recruit caspase-1, which can cleave pro-IL-1β and pro-IL-18 to mature forms that participate in the innate immune response [6, 7].

The pathological mechanisms for NLRP3 inflammasome activation and downstream cytokine production in T2DM have been elucidated. Indeed, inflammatory cytokines are crucial pathophysiological determinants of T2DM, as activated IL-1β and IL-18 can cause islet β cell injury/death and dysfunction by inducing inflammationand immune cell infiltration. Insulin resistance or suppression of sensitivity then occurs, which leads to T2DM [8–10]. Emerging evidence suggests that newly diagnosed T2DM patients show significantly upregulated protein expression of NLRP3, ASC, caspase-1, IL-1β and IL-18 among monocyte-derived macrophages [11]. Furthermore, inhibition of caspase-1 and IL-1β receptors can augment insulin sensitivity and ameliorate the secretory function of β cells [12].

The relationship between AS and NLRP3 inflammasome activation and downstream cytokine production is ambiguous. IL-1β and IL-18 are two multifunctional cytokines involved in chronic inflammation that induce an intense inflammatory response, which is important in the pathogenesis and progression of AS [13]. Recent studies have demonstrated that atherogenesis can be initiated through the NLRP3 inflammasome pathway triggered by endogenous molecules, such as crystalline cholesterol [14]. Menu P et al. confirmed that the absence of NLRP3, ASC and capase-1, the core constituents of the NLRP3 inflammasome, did not affect AS progression in an apolipoprotein E (ApoE)-deficient mouse model [15]. Duewell P and coworkers [16] found contrasting results using a low-density lipoprotein receptor (LDLR)-deficient mouse model transplanted with NLRP3-, ASC-, or IL-1β-deficient cells.

Because little is known about the NLRP3 inflammasome, IL-1β and IL-18 in patients with T2DM and AS, here we performed a cross-sectional study investigating these inflammatory components in patients with T2DM complicated with carotid atherosclerosis (CAS).

## Methods

### Research objects

In this cross-sectional study, a total of 107 inpatients or outpatients at the Jingzhou Affiliated Hospital of Huazhong Technology University were enrolled, including 81 T2DM + CAS patients (37 cases with increased intima media thickness (IMT) and 44 cases with plaque) and 26 T2DM patients. T2DM was diagnosed according to the American Diabetes Association (ADA) guidelines, defined as either (1) a fasting plasma glucose level of ≥126 mg/dL, (2) a 2-h plasma glucose level of ≥200 mg/dL after a 75-g oral glucose tolerance test, or (3) a glycated hemoglobin level of ≥6.5%. T2DM was confirmed by a result above the diagnostic threshold in two out of the three above-mentioned tests. Patients who were receiving antidiabetic medications (oral hypoglycemia agents and/or insulin) were deemed to have T2DM. All enrolled individuals showed no evidence of recent acute or chronic infectious disease, patients who have CVD including cerebral infarction, myocardial infarction (MI), angina, heart failure were also excluded. All examinations were permitted by participants and approved by the Ethics Committee of Jingzhou Central Hospital, Affiliated Tongji Medical College, Huazhong University of Science and Technology. Written consent was obtained from all participants according to the Declaration of Helsinki.

### Measurement of carotid plaque size and IMT

The bilateral common carotid artery, carotid bifurcation, and internal and external carotid artery of participants were scanned using duplex ultrasonography with a 10-MHz transducer (ie33, Ultrasound Imaging Systems, Holland). Plaque thickness was recorded and scored using the Crouse method [17]. If no plaque was found, the IMT at one site away from sinus in the common carotid artery was detected. Participants with an IMT > 1.5 mm were enrolled in the plaque group, whereas those with an IMT ≥ 1.0 mm and ≤1.5 mm were enrolled in the IMT thickened group.

### Measurement of serum IL-1βand other factors

Serum IL-1β and IL-18 concentrations were measured using commercially available enzyme-linked immunosorbent assay (ELISA) kits (TSZ Biotech, USA). Prior to sample detection, a standard curve was established with a correlation coefficient of 0.998. The height, weight, blood pressure, peripheral blood leukocyte amount, mononuclear cell count, fasting blood glucose (FBG), serum triglyceride (TG), total cholesterol (TC), high-density lipoprotein (HDL) and low-density lipoprotein (LDL) were also measured.

### Measurement of NLRP3 pathway genes

1, RNA isolation and reverse transcription in: Fresh peripheral blood was obtained and centrifuged and then stratified using a Ficoll-Paque Plus density gradient (20,130,613, Solarbio Science, Beijing) according to the manufacturer’s protocol. Peripheral blood monocytes cells (PBMCs) were acquired, and total RNA was isolated using TRIzol Reagent (14,105, Life Technologies, America). RNA was reverse transcribed to cDNA usingPromega MMLV reverse transcriptase (00021405, Gold Star, Beijing) according to the manufacturer’s protocol. 2, Real-time fluorescence quantitative PCR: cDNA was amplified by real-time PCR with gene-specific primers (from Life Technologies). The constituents of the real-time PCR were added according to the manufacturer’s protocol. The reaction conditions were as follows: 95 °C for 10 min;10 amplification cycles of 95 °C for 30s, 56 °C for 30 s, and 62 °C for 45 s; and then 30 amplification cycles of 95 °C for 15 s, 56 °C for 15 s, and 62 °C for 45 s. The PCR amplification products were verified by electrophoresis on 1.5% agarose gel and stained with ethidium bromide.

mRNA expression levels are described as △△Ct (cycle threshold) values relative to the controls. Duplicate samples were subjected to RT-PCR, and the mean was used for analysis. The primers used for each gene were designed with Primer Express v2.0 (v. 0.4.0) andarelisted in Table 1.

**Table 1:** Primers for NLRP3 inflammasome component genes

	forward primer	reverse primer	probe
NLRP3	TGCCCGTCTG GGTGAGA	CCGGTGCTCCTTGATGAGA	TGAGCCTCAACAAACGCTAC ACACGACT
ASC	CGCGAGGGTC ACAAACGT	TGCTCATCCGTCAGGACCTT	AGTGGCTGCTGGATGCTCTG TACGG
caspase-1	AATTTTCCGCAAGGTTCGAT T	ACTCTTTCAGTGGTGGGCAT CT	TCATTTGAGCAGCCAGATGG TAGAGCG
β-actin	GCTGTCTGCCTTGGTAGTGG AT	GCATCGTCACCACCAAAGC	ATGGGTCAGGGATGTGCAAG GCA

### Statistics

All data were analyzed with SPSS 13.0. Data with normal distribution are presented as mean ± standard deviation and those with skewed distribution are presented as median(P_25_~P_75_). Fold change (2-△△Ct) in mRNA expression were calculated relative to control subjects. Student’s t test or Mann-Whitney U test were used for comparison on continuous variables between two groups, The frequency data between two groups were compared by chi-squared test. All statistic test were two-tailed, A *p* value <0.05 was considered statistically significant.

## Results

### Basic characteristics of subjects

Table 2 shows the demographic information for the T2DM and T2DM + CAS groups. No significant differences were observed regarding gender, drinking, smoking, FBG,TG, HDL, APOA between the two groups (*p* > 0.05). The ages of T2DM + CAS groups were higher than T2DM group (*p* < 0.05); however, there is no report indicating that age influences the expression of NLRP3 pathway genes and secretion of IL-1β and IL-18. The percentages of hypertension was also higher than T2DM group (*p* < 0.05), which is not surprising [18]. CHOL、LDL were higher in T2DM + CAS group than T2DM group (*p* < 0.05). Table 3 shows demographic information for the T2DM + AS patients with plaque or not. No significant differences were observed in the sex ratio, drinking, smoking, hypertension incidence or FBG between the IMT thicken and plaque group (*p* > 0.05).

**Table 2:** Characteristics of T2DM patients combined with CAS or not

Variables	T2DM, *n* = 26	T2DM + CAS, *n* = 81	*P*
Female, n (%)	13 (50.0)	31 (38.3)	0.290
Drinking, n (%)	3 (11.54)	20 (24.69)	0.155
Smoking, n (%)	9 (34.62)	30 (37.04)	0.823
Hypertension, n (%)	3 (11.54)	43 (53.09)	<0.001
Age(year), mean ± sd.	51.42 ± 10.2	59.12 ± 12.19	0.004
Leukocyte(10^9^/L), mean ± sd.	5.49 ± 1.08	6.46 ± 2.07	0.046
Mononuclear(10^9^/L), mean ± sd.	2.88 ± 0.77	3.96 ± 2.02	0.014
FBG(mmol/l), mean ± sd.	11.71 ± 3.96	10.31 ± 3.27	0.095
TG(mmol/l), mean ± sd.	2.57 ± 2.72	2.26 ± 1.68	0.681
CHOL (mmol/l), mean ± sd.	4.54 ± 1.07	5.03 ± 1.28	0.043
HDL(mmol/l), mean ± sd.	1.01 ± 0.23	1.06 ± 0.24	0.487
LDL(mmol/l), mean ± sd.	2.56 ± 0.76	3.03 ± 1.02	0.014
APOA(g/L), mean ± sd.	1.35 ± 0.2	1.31 ± 0.22	0.468
APOB(g/L), mean ± sd.)	0.88 ± 0.24	1.03 ± 0.29	0.022
LPa(mg/L), mean ± sd.	100.19 ± 103.77	148.89 ± 176.1	0.085

**Table 3:** Characteristics of T2DM + CAS patients with plaque or not

Variables	IMT thicken, *n* = 32	Plaque, *n* = 49	*P*
Female, n (%)	13 (40.63)	18 (36.73)	0.725
Drinking, n (%)	10 (31.25)	10 (20.41)	0.269
Smoking, n (%)	12 (37.50)	18 (36.73)	0.944
Hypertension, n (%)	13 (40.63)	30 (61.22)	0.069
Age(year), mean ± sd.	52.34 ± 11.01	63.55 ± 10.9	<0.001
Leukocyte(10^9^/L), mean ± sd.	6.6 ± 2.58	6.36 ± 1.65	0.865
Mononuclear(10^9^/L), mean ± sd.	4.11 ± 2.48	3.86 ± 1.65	0.848
FBG(mmol/l), mean ± sd.	10.56 ± 2.70	10.15 ± 3.61	0.223
TG(mmol/l), mean ± sd.	2.53 ± 2.13	2.09 ± 1.30	0.823
CHOL (mmol/l), mean ± sd.	5.04 ± 1.28	5.02 ± 1.30	0.924
HDL(mmol/l), mean ± sd.	1.10 ± 0.24	1.04 ± 0.25	0.301
LDL(mmol/l), mean ± sd.	3.07 ± 1.11	3.01 ± 0.98	0.932
APOA(g/L), mean ± sd.	1.35 ± 0.24	1.29 ± 0.21	0.305
APOB(g/L), mean ± sd.	1.05 ± 0.30	1.01 ± 0.29	0.621
LPA(mg/L), mean ± sd.	152.07 ± 160.76	146.83 ± 187.14	0.770

Partial data are expressed as mean ± standard deviation. “a” represent the *p* value <0.05 compared with control; “b” represent the *p* value <0.05 compared with T2DM + CAS. Y/N, yes/no; BMI, body mass index; T2DM + CAS, type 2 diabetes mellitus complicated with carotid atherosclerosis; T2DM, type 2 diabetes mellitus; CAS, carotid atherosclerosis; FBG, fasting blood glucose; TG, triglyceride; TC, total cholesterol; HDL, high-density lipoprotein; LDL, low-density lipoprotein.

### Comparison of NLRP3 inflammasome component mRNA expression and IL-1β and IL-18 production

We used the 2^-△△Ct^ (Ct, cycle threshold) method to assess changes in mRNA expression. Table 4 denotes the mRNA expression levels of NLRP3 inflammasome component genes (NLRP3, ASC, and capase-1) in the T2DM and T2DM + CAS groups. The expression levels of NLRP3 and ASC mRNA were increased significantly in T2DM + CAS group compared with T2DM group (*p* < 0.05). However, no difference was observed in caspase-1, serum IL-1β and IL-18 concentrations in T2DM and T2DM + CAS group (*p >* 0.05).

**Table 4:** NLRP3, ASC, Caspase-1, IL-1 and IL-18 level in T2DM patients combined with CAS or not

Variables	T2DM, *n* = 26	T2DM + AS, *n* = 81	*P*
NLRP3 mRNA, mean ± sd.	1.74 ± 0.81	3.38 ± 1.74	<0.001
ASC mRNA, mean ± sd.	2.07 ± 0.92	2.48 ± 0.61	0.033
Caspase-1 mRNA, mean ± sd.	2.86 ± 2.17	3.64 ± 1.80	0.065
IL-1β(pg/ml), mean ± sd.	56.06 ± 36.97	54.87 ± 26.81	0.561
IL-18(pg/ml), mean ± sd.	158.29 ± 88.96	149.2 ± 67.96	0.996

Data are expressed as mean ± standard deviation. “a” represent the *p* value < 0.05 compared with control; “b” represent the *p* value < 0.05 compared with T2DM + CAS. IL-1β, interleukin-1β; IL-18, interleukin-18. When divided T2DM + CAS group into IMT thickened and plaque groups, as shown in Table 5, the expression levels of the three inflammasome genes and IL-18 were increased compared with the plaque group (*p* < 0.05). The IL-1β level shows no difference between the two groups.

**Table 5:** NLRP3, ASC, Caspase-1, IL-1 and IL-18 level in T2DM + AS patients with plaque or not

Variables	IMT thicken, *n* = 32	Plaque, *n* = 49	*P*
NLRP3 mRNA, mean ± sd.	3.84 ± 1.51	2.95 ± 1.20	0.004
ASC mRNA, mean ± sd.	2.76 ± 0.51	2.21 ± 0.39	<0.001
Caspase-1 mRNA, mean ± sd.	4.39 ± 1.63	2.94 ± 1.74	<0.001
IL-1β(pg/ml), mean ± sd.	60.48 ± 35.01	51.51 ± 20.19	0.682
IL-18(pg/ml), mean ± sd.	186.57 ± 73.47	126.77 ± 53.88	0.003

Partial data are expressed as mean ± standard deviation

### Correlation between NLRP3 inflammasome mRNA expression, downstream cytokinesons and the Crouse scores

**Fig. 1:**
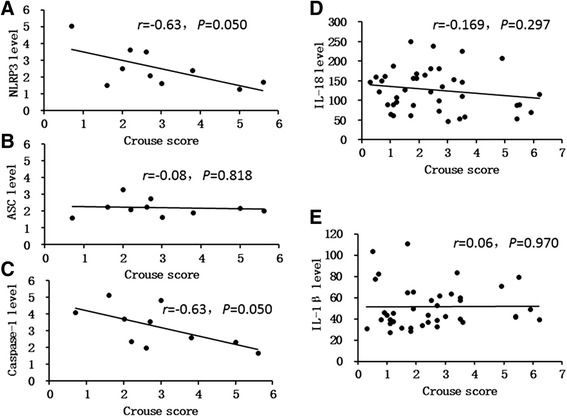
Correlation between NLRP3 inflammasome component genes expression, downstream cytokinesons and the Crouse scores. **a**, **b** and **c** NLRP3 mRNA、ASC mRNA、Caspase-1 mRNA expression in PBMCs of T2DM + CAS patients show negative correlation to Crouse scores separately. **d** and **e** Serum IL-18 concentrations were negatively correlated with the Crouse scores evidently, but Serum IL-1β concentrations were not

Forty-four participants had plaque in the T2DM + CAS group, and the mean Crouse score in this group was 4.90 ± 3.01. The mRNA expression levels of NLRP3 inflammasome pathway genes and serum IL-18 concentrations were negatively correlated with the Crouse scores; the correlation coefficients are shown in Fig. 1.

## Discussion

Diabetes and AS are metabolic diseases with a high global incidence. Diverse reports have suggested that metabolic diseases are associated with chronic inflammation, in which NLRP3 inflammasome activation and IL-1β and IL-18 cytokine production have been implicated [19–21]. Thus, assessing mRNA expression of NLRP3 inflammasome genes in vivo in various diseases in humansis essential to fully clarify the mechanisms of pathogenesis and determine targeted therapy for inflammatory diseases.

Recent studies have shown that NLRP3, ASC and caspase-1 expression and IL-1β and IL-18 secretion are increased under high-glucose conditions in vivo and in vitro [11, 22, 23]. Additionally, upregulated expression of inflammasome components and downstream cytokines was demonstrated in atherosclerotic patients and animal models [24, 25]. In our study, results in Table 4 shows that NLRP3 mRNA and ASC mRNA were increased significantly in T2DM patients and CAS patients compared with T2DM patients (*p <* 0.05) corroborate these previous findings, excluding the caspase-1 expression levels which possibly due to negative regulation of the NLRP3 pathway [7, 26, 27]. In addition, serum IL-18 concentrations in the T2DM group and T2DM + CAS group shows no difference(*p* > 0.05). The many influencing factors of IL-18 levels in blood make it difficult to interpret these results, and further studies are needed to investigate additional mechanisms.

There are few reports on the NLRP3 inflammasome in patients with diabetes with CAS. LI Y et al. [28] suggested merely that NLRP3 inflammasome markers were increased in diabetic pigs with atherosclerotic lesions compared with control pigs. Here, we investigated the mRNA expression levels of NLRP3 and ASC from T2DM + CAS patients were significantly higher than purely diabetic patients (*p* < 0.05). AS we know, NLRP3 can detect endogenous danger signals, such as high glucose, fatty acid, uric acid, cholesterol crystals, and bacteria, and cholesterol crystals are considered the primary activator of NLRP3 during atherosclerotic plaque formation [2, 13, 29, 30]. IL-1β and IL-18 are downstream cytokines of the NLRP3 inflammasome, and their serum concentrations are predictors of AS and T2DM [20]. Thus, under conditions of high glucose in the presence of cholesterol crystals, the inflammatory immune response is greater. So, the NLRP3 inflammasome pathway can be synergistically activated by hyperglycemia and AS, with consequently increased mRNA expression of NLRP3, ASC and caspase-1.

Then T2DM + CAS patients in our study were divided into IMT thickened and plaque groups. The measurement of IMT or plaque by ultrasonography is essential to detect prevalent cardiovascular diseases [31]. Increased IMT is considered an early event in the formation of plaques or AS. The mRNA expression levels of NLRP3 inflammasome component genes and the serum IL-18 levels in the IMT thickened group were significantly increased compared to those in the plaque group (*p* < 0.05), demonstrating that the inflammatory response mediated by the NLRP3 inflammasome was more active at the early stage of plaque formation. At this stage, vascular lesions cause macrophage infiltration, foam cell formation and lipid deposits, followed by lipid accumulation and increased IMT and persistent NLRP3 inflammasome activation. With plaque progression, the fibrous cap forms, and mixtures containing cholesterol clefts are produced. Thus, it is possible that the response of NLRP3 inflammasome diminished due to the consumption of these cholesterol substances at the mid-advanced stage of plaque formation [27, 32, 33]. Many immune cells can be detected in subendothelial areas in the early stage of AS plaque formation [34], and IL-1β and IL-18 activation is considered the core element of AS formation [35]. As shown in Table 5, NLRP3, ASC, caspase-1 and serum IL-18 levels in the IMT thickened group were higher than those in the plaque group, which also shows that the downstream cytokines of the NLRP3 inflammasome were more active at the early stage of plaque formation.

The present study also explored the relationship between Crouse plaque scores and the mRNA expression levels of NLRP3, ASC, caspase-1 and serum IL-1β and IL-18 concentrations in the plaque group. The Crouse score is used to evaluate the AS plaque burden and is associated with CAS severity [36]. As shown in Fig. 1, the Crouse score was negatively correlated with these factors. These data further confirm that the NLRP3 inflammasome pathway is more active at the early stage of plaque formation in T2DM + CAS patients.

From the investigation, we found that the degree of activation of the NLRP3 inflammasome pathway was higher when AS coincided with T2DM, with greater activity observed at the early stage of plaque formation in this study. However, the combination of T2DM and CAS is a complex pathological phenomenon that requires further research, such as gene knockout experiments or histopathology, to reveal potential mechanisms responsible for disease development and progression [32].

## Conclusion

In this study, we uncover the inflammasome pathway of NLRP3 is remarkably increased in patients with AS and T2DM at the early stage of plaque formation. The current study has some limitations. First, our study is a cross-sectional study, the number of subjects and selected diseases were limited. The second limitation is that only part of the experiment index and diabetic patients with carotid artery were observed. However, our findings of NLRP3 inflammasome pathway activity is significantly increased in patients with AS and T2DM at the early stage of plaque formation provides a potential therapeutic strategy to treat these patients. In the future studies, the large prospective study, demonstration of related gene knockout in animal model and the study of anti-inflammatory cytokines, warrant further investigation.

## Abbreviations

ADA: American Diabetes Association
ApoE: AS progression in an apolipoprotein E
AS: Atherosclerosis
CAS: Carotid atherosclerosis
FBG: Fasting blood glucose
HDL: High-density lipoprotein
IL-18: Interleukin-18
IL-1β: Interleukin-1β
IMT: Intima media thickness
LDL: Low-density lipoprotein
LDLR: Low-density lipoprotein receptor
MI: Myocardial infarction
NLRP3: Nucleotide-binding domain, leucine-rich-containing family, pyrin domain-containing-3
T2DM + CAS: T2DM complicated with carotid atherosclerosis
T2DM: Type 2 diabetes mellitus
TC: Total cholesterol
TG: Serum triglyceride
